# Improved pulse wave velocity wave front detection by template-matching

**DOI:** 10.1186/1532-429X-16-S1-P361

**Published:** 2014-01-16

**Authors:** Travis Sharkey-Toppen, Yu Ding, Bradley D Clymer, Orlando P Simonetti, Subha V Raman

**Affiliations:** 1 The Ohio State University, Columbus, Ohio, USA

## Background

Pressure waves distributed through the aorta by left ventricular contraction propagate at a rate known as the pulse wave velocity (PWV). PWV is a measure of aortic stiffness, and increased PWV has been correlated with increased coronary artery disease risk. PWV can be measured by velocity-encoded magnetic resonance (PC-MR), with velocity profiles collected at a minimum of two locations along the vessel. The commonly used simple linear regression method computes PWV from detected wavefronts in each profile and distance along the aorta. Recognizing the limitation imposed on wave front detection by simple maximum impulse by noise in the signal producing local maxima, we designed and tested a template-matching scheme to improve PWV estimation.

## Methods

**Figure 1:**
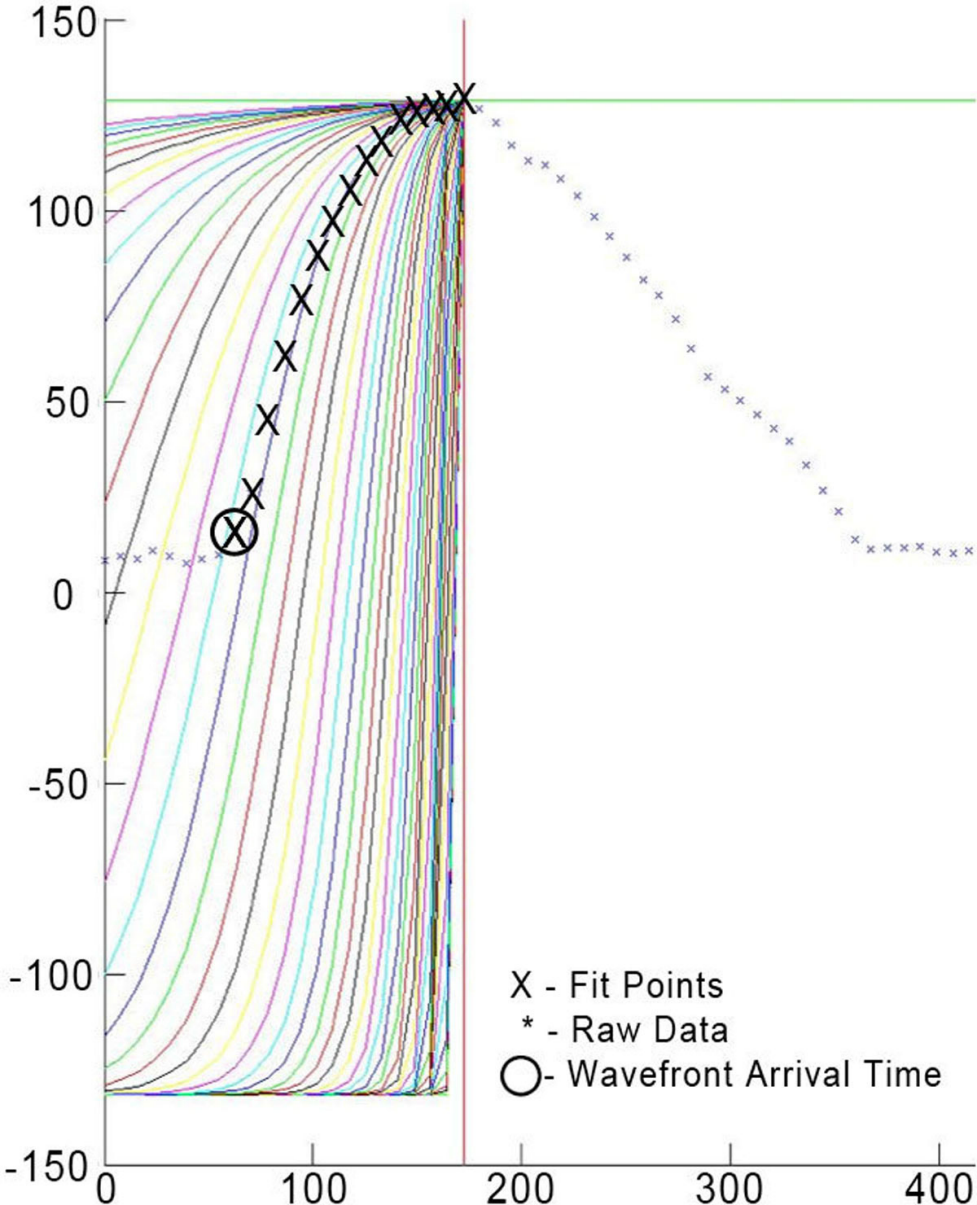
**Example of template-matching raw data to an optimized generalized logistic function**. Each curve varies the control parameter, a, until the fit is maximized.The circled center point is then chosen as the wavefront arrival time for the given distance.

In vivo CMR was performed on a 3T scanner (Verio, Siemens Healthcare) in perimenopausal women with a variable number of atherosclerosis risk factors but no evident atherosclerotic disease. The derivation cohort consisted of 12 scans (4 patients over 3 time points) and validation cohort consisted of 139 scans PC-MR was acquired in an oblique sagittal plane through the descending aorta with in-plane velocity encoding in the head-foot direction and prospective ECG triggering (TE/TR2.1/9.15 ms, Venc 150 cm/s, flip angle 15°). Distance from the base of the aorta and velocity were collected along the centerline of the aorta. Wave detection was performed at each cross section by the following scheme. A generalized logistic function f(a,t) = -Vmax+2Vmax/[1+exp{-a(t-c)}], c = tmax+ln(1/.99-1)/a, the center point, where Vmax is the maximum velocity, t is time, tmax is the time of the observed Vmax, and a was optimized to maximize the goodness of fit between the template and observed data between c and tmax. An example fit varying the control parameter, a, is shown in Figure 1. Pulse wave velocity was then calculated using simple linear regression over the (c, distance) pairs collected along the aorta. A one-tailed Student t-test with α of .05 was used to test for a significant increase in goodness of fit.

## Results

**Figure 2:**
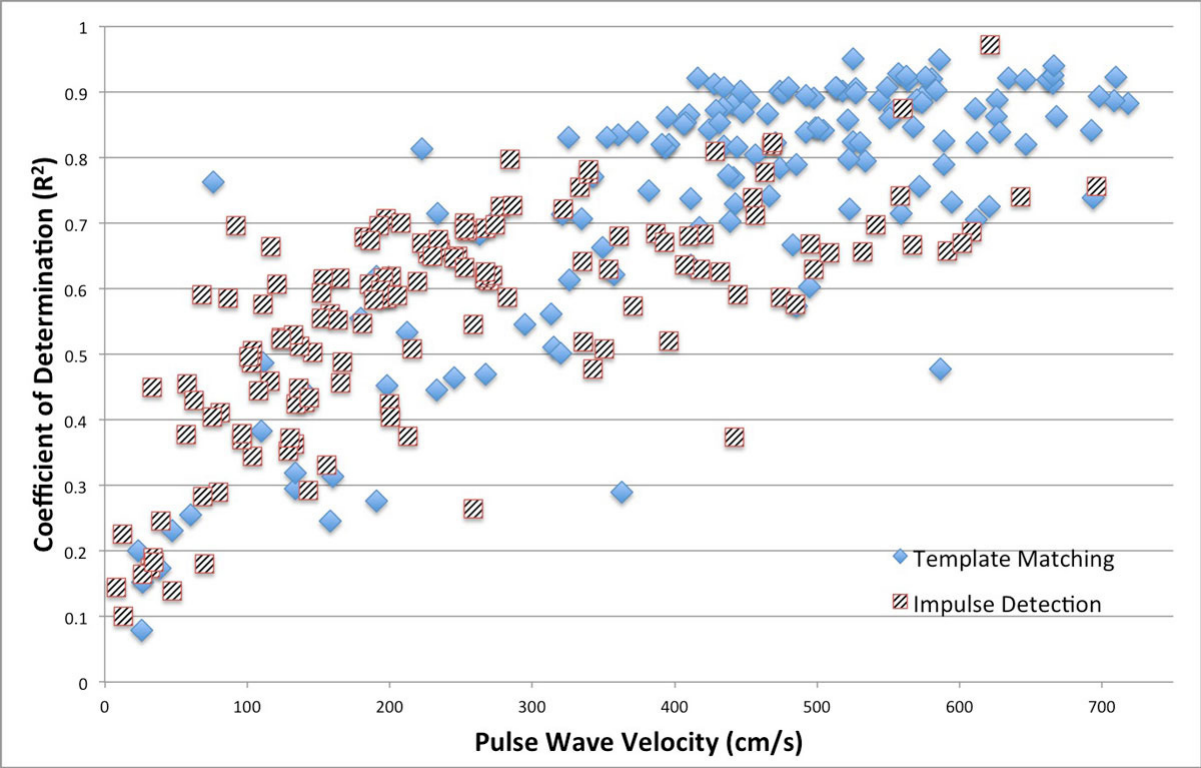
**Scatter plot of the coefficient of determination for each estimation versus the calculated pulse wave velocity for both wavefront detection methods**. Template-matching demonstrates an overall improved goodness of fit (p < 0.0001).

The proposed method for estimation of pulse wave velocity yielded a 53% improvement in overall goodness of fit (p < 0.0001) when compared to estimations using maximum impulse detection (Figure 2).

## Conclusions

A template-matching scheme provides more robust estimation of aortic PWV compared to maximum impulse detection. This approach warrants incorporation into longitudinal studies of cardiovascular risk and aortic pathophysiology to improve reliability of PWV as an outcome measure.

## Funding

NIH R01HL095563.

